# *In vitro* and *in vivo* antimicrobial effects of domiphen combined with itraconazole against *Aspergillus fumigatus*

**DOI:** 10.3389/fmicb.2023.1264586

**Published:** 2023-11-21

**Authors:** Huan Yang, Yufan Gu, Zhiqiang He, Jia-Nan Wu, Chen Wu, Yuhan Xie, Yuxin You, Yijia Yang, Xinyu Zhou, Yi Liu, Wenlong Du

**Affiliations:** ^1^Department of Bioinformatics, School of Life Sciences, Xuzhou Medical University, Xuzhou, Jiangsu, China; ^2^Department of Biophysics, School of Life Sciences, Xuzhou Medical University, Xuzhou, Jiangsu, China

**Keywords:** domiphen, itraconazole, *Aspergillus fumigatus*, synergistic effect, azole resistance

## Abstract

*Aspergillus fumigatus*, a prevalent saprophytic fungus in the atmosphere, is known to rapidly induce severe invasive aspergillosis (IA) upon inhalation of its conidia by humans or animals. The mortality rate associated with IA exceeds 50%. The misuse of antifungal agents has contributed to the emergence of numerous highly pathogenic drug-resistant strains of *A. fumigatus*. Our study found that the combination of domiphen and itraconazole had sound synergistic antimicrobial effects against wild-type and itraconazole-resistant *A. fumigatus in vivo* and *in vitro* through MIC, FIC, plate inoculation, growth curve experiments, and *Galleria mellonella* infection model. Drug cytotoxicity and pharmacological tests for acute toxicity assays demonstrated that both itraconazole and domiphen showed minimal cytotoxicity and good biocompatibility. The transcriptome sequencing experiment demonstrated that domiphen exerted a suppressive effect on the expression of various genes, including those involved in drug efflux, redox regulation, and cellular membrane and cell wall remodeling. The present investigation explores the synergistic antimicrobial mechanisms of domiphen and itraconazole, encompassing three key aspects: (i) domiphen inhibited the efflux of itraconazole by reducing the expression of drug efflux-related genes, (ii) the combination has good ability to disrupt the cell membrane and cell wall, (iii) the combination also can remove biofilm more effectively. In summary, the utilization of domiphen as a synergist of itraconazole exhibited disruptive effects on the biofilm, cell wall, and cell membrane of *A. fumigatus*. This subsequently led to a modified distribution of itraconazole within the fungal organism, ultimately resulting in enhanced antifungal efficacy. The results of this study may provide a new therapeutic strategy for the treatment of IA caused by drug-resistant *A. fumigatus*.

## 1 Introduction

In immunocompromised individuals, Invasive Aspergillosis (IA) is the prevailing fungal infection, primarily attributed to *Aspergillus* spp. This filamentous fungus can initiate infection upon inhalation of its conidia (El-Baba et al., 2020; Cho et al., 2022). Over the past years, there has been a notable rise in the occurrence of IA and drug resistance, resulting in hundreds of thousands of cases annually that pose a significant threat to life, with a mortality rate surpassing 50% (Cadena et al., 2021; Bosetti and Neofytos, 2023). *Aspergillus fumigatus* is a prevalent etiological agent of invasive fungal infections, characterized by its rapid proliferation and ability to adapt to adverse growth conditions. It frequently colonizes the pulmonary system, leading to clinical manifestations resembling tuberculosis in affected individuals (Keizer et al., 2020; Suleyman and Alangaden, 2021).

Currently, the most efficacious antifungal medications encompass polyenes, triazoles, echinocandins (Van Daele et al., 2019; Punia et al., 2022). These drugs exert their inhibitory effects by binding to ergosterol, thereby impeding cell membrane formation, or by targeting the synthesis of ergosterol and β-1,3 glucan, which hinders cell wall and cell membrane formation (Pierard et al., 2012). In clinical settings, triazoles are frequently favored. Nevertheless, the extensive utilization of these medications has led to the gradual emergence of drug resistance in fungi, posing a substantial obstacle to the treatment of IA (Ben-Ami and Kontoyiannis, 2021; Denning, 2022; Kaur and Nobile, 2023; Meng et al., 2023).

In recent years, J.-P. Latgé et al. have put forth three primary approaches for the treatment of aspergillosis, namely combination drug therapy, surgical intervention, and immunotherapy (Latge and Chamilos, 2019). Among these strategies, combination drug therapy has gained significant recognition due to its support from *in vitro* data and clinical studies, making it a pivotal advancement in addressing azole drug resistance caused by *A. fumigatus* (Zeng et al., 2019; Murphy and Bicanic, 2021). Itraconazole, a potent triazole-class antifungal drug with broad-spectrum activity, effectively inhibits ergosterol synthesis in fungal cell membranes, resulting in the accumulation of methyl sterols (Negroni and Arechavala, 1993). This imbalance of sterol components disrupts membrane integrity, inhibits fungal growth, and even kills fungi. It has proven to be a valuable option to the currently available antifungal drugs for treatment. In addition, domiphen is a cationic surface-active agent broad-spectrum fungicide that readily adsorbs to the surface of the fungus, altering the permeability of the fungal cytoplasmic membrane and disrupting its metabolism to produce antifungal effects (Tits et al., 2020a,b; Long et al., 2021). Combining the two drugs may produce a synergistic effect and thus serve as a new strategy for antimicrobial drugs and clinical treatment (Dong et al., 2021).

The objective of this study was to investigate the potential synergistic antimicrobial effect of combining itraconazole with domiphen against azole drug-resistant *A. fumigatus in vivo* and *in vitro*, as well as to explore the underlying mechanism of this synergistic effect in order to enhance the treatment of IA caused by azole drug-resistant strains. The findings from this research will offer a novel therapeutic strategy for managing IA associated with azole drug resistance.

## 2 Materials and methods

### 2.1 Reagents

Itraconazole (98%) and domiphen (97%) were procured from Shanghai Macklin Biochemical Co., Ltd. (China). The HT-22 cells and HK-2 cells were purchased from Shanghai Gaining Biological Co., Ltd. (China). Propidium Iodide was obtained from Xian Runxue Bio-Technology Co., Ltd. (China). Phosphate Buffer Saline (PBS) was acquired by Jiangsu Keygen Biotech Co., Ltd. (China).

### 2.2 Preparation of strains

This study examined a total of four strains, namely *A. fumigatus* 1161 (wild type), Cox 10 (laboratory extract), Shjt 40, and Shjt 42b (extracted from clinical patients), with the latter three exhibiting resistance to azole (Wei et al., 2017; Du et al., 2021). *A. fumigatus* was preserved in 50% glycerol at a temperature of −20°C. Unless otherwise stated, the concentration of fungal suspensions utilized in subsequent experiments was 1 × 10^6^ CFU/mL.

### 2.3 Drug cytotoxicity

Adherent cells (HT-22 and HK-2) in the log phase were digested by adding trypsin, and digestion was terminated by adding a serum-containing medium. The cells were then transferred to a centrifuge tube and centrifuged (1,000 r/min, 5 min). The supernatant of the centrifuge tube was discarded and resuspended with a serum-containing medium. The cells were counted using a cell counter and diluted to 10^5^ cells/ml. 96-well plates were taken, and 100 μL of diluted cell suspension was added to each well of the plate. A total of seven experimental groups, consisting of one blank control and six parallel controls, were established. Following 24 h of incubation, the medium was aspirated. Subsequently, different concentrations of itraconazole and domiphen were added to the 96-well plates. After an additional 24 h of incubation, the drugs were aspirated, and the medium containing cck-8 was added and incubated for a duration of 2 h. Finally, the samples were subjected to observation under a microplate reader at a wavelength of 450 nm. The absorbance of the experimental group is expressed as Aa. The absorbance of the control group and the blank group were expressed as Ac and Ab, respectively (Wen et al., 2022).

$$\mathrm{Ratio}\;\mathrm{of}\;\mathrm{cell}\;\mathrm{survival}=\left(\mathrm{Aa}-\mathrm{Ab}\right)/\left(Ac-\mathrm{Ab}\right)\;x\;100\%$$

### 2.4 Pharmacological tests for acute toxicity

5 zebrafishes were put into a 500 mL water environment resembling their feeding conditions. Subsequently, the appropriate concentration of drugs was added, and three parallel control groups were established. The growth (or mortality) of the zebrafish was observed and recorded after a 24-h period. The minimum drug concentration at which the number of deceased individuals exceeded half of the total number of experimental subjects was designated as LC50. This numerical value serves as an indicator of the drug’s toxicity toward the zebrafish population (Nikam et al., 2020).

### 2.5 Single-drug sensitivity

The minimum inhibitory concentration (MIC) was determined using the methodology established by the Clinical and Laboratory Standards Institute (CLSI document M38-A2; Espinel-Ingroff et al., 2011) with minor modification. Briefly, we prepare several centrifuge tubes with MM (minimal medium) instead of RPMI-1640 medium as solvent to obtain itraconazole solutions of different concentrations according to our previous reports demonstrating that these two media have no influence on the experimental result (Du et al., 2023). And then, 20 μL of the diluted fungal solution was inoculated into the centrifuge tubes and incubated at 37°C in an incubator for 36 h. The MIC of itraconazole on *A. fumigatus* was determined by comparing the turbidity of the liquid in each tube, and the lowest itraconazole concentration without fungal growth (clear) was the MIC of itraconazole. The MIC of domiphen on *A. fumigatus* was tested as above.

### 2.6 Colony inhibition

The growth of *A. fumigatus* on the plates was used to determine the effect of different drugs and different concentrations on *A. fumigatus*, and the inhibition effect of the drugs was detected by the diameter of *A. fumigatus* colonies. Suspensions of *A. fumigatus* at concentrations of 1 × 10^8^ CFU/mL, 1 × 10^7^ CFU/mL, and 1 × 10^6^ CFU/mL were extracted and thoroughly mixed. A volume of 2 μL of *A. fumigatus* suspension was aspirated, starting from the highest concentration and moving to the lowest, and inoculated onto the solid medium containing different concentrations of drugs, resulting in a suspension drop approximately 2 mm in diameter. The inoculated medium was then placed in a thermostat set at 37°C and incubated for a duration of 36 h (Du et al., 2023).

### 2.7 Determination of antifungal activity

Further analysis was conducted to determine the antifungal activity of the combination of domiphen and itraconazole, based on the obtained minimum inhibitory concentrations (MICs) of each drug. 1/2 × MIC itraconazole, 1/2 × MIC domiphen, and a combination of 1/4 × MIC domiphen with 1/4 × MIC itraconazole were prepared using liquid MM as a solvent. The solution was then inoculated with a diluted fungal suspension, and at least 6 parallel controls were established and incubated. Following a 24-h incubation period, the biomass of the fungus was assessed using CFU counting. The cultures underwent washing and dissolution in PBS, and subsequent serial dilutions were applied onto MM agar plates. The plates were then incubated at 37°C for 48 h, after which the colonies were counted (Tits et al., 2020b).

### 2.8 Microbial growth curve

The single drug treatment group was itraconazole or domiphen diluted with sterile MM to a concentration of 1/2 × MIC. The combined drug group was 1/4 × MIC itraconazole with 1/4 × MIC domiphen, and the diluted drug was added to a 96-well plate with a blank control (no drug was added in MM), and six parallel controls were taken. 20 μL of *A. fumigatus* was added and subsequently the biomass of *A. fumigatus* was recorded at OD600 hourly and plotted as a growth curve (Furukawa et al., 2020).

### 2.9 Combined drug sensitivity

Itraconazole and domiphen were diluted with sterile MM at concentrations starting at 8 × MIC, using the two-fold dilution method. Each 96-well plate was treated with 50 μL of itraconazole and 50 μL of domiphen, along with 100 μL of fungal solution. The plates were then incubated at 37°C for 36 h. The lowest drug concentration (apparently clear) without microbial growth was MIC’. The specific formula for FIC is given below (Xu et al., 2018). MIC and MIC’ represent the MIC of single drug and the MIC of combined drug, respectively.

$$\begin{array}{l} FIC={\mathrm{MIC}}^{’}\;\left(\mathrm{Itraconazole}\right)/\mathrm{MIC}\;\left(\mathrm{Itraconazole}\right)\\ +\;{\mathrm{MIC}}^{’}\;\left(\mathrm{Domiphen}\right)/\mathrm{MIC}\;\left(\mathrm{Domiphen}\right) \end{array}$$

### 2.10 *In vivo* antifungal effect

A total of 120 *G. mellonella* larvae were selected for the experiment. The larvae were divided into eight groups: normal saline control group, domiphen injection group, itraconazole injection group, combined injection group, *A. fumigatus* injection group, domiphen injection group after *A. fumigatus* infection, itraconazole injection group after *A. fumigatus* infection, and combined injection group after *A. fumigatus* infection. Injection site is the second foot from the lower right. The larvae, which were subjected to fungal infection at a concentration of 10^6^ CFU/mL, require administration of the drug 1 h subsequent to the initial injection. The concentration of all administered drugs was maintained at 2 MIC, while the volume of all injected reagents was 10 μL. The mortality of the larvae was monitored and documented every 24 h, and a mortality curve was generated (Sun Y. et al., 2022).

### 2.11 mRNA sequencing

The heatmap showing log_2_FC of each genes was generated using MeV based on RNA-seq data shown in Supplementary Table 1 (Du et al., 2023).

### 2.12 Extracellular drug content assay

A centrifuge tube containing 1 mL of MM medium was inoculated with 1 mL of diluted fungal solution and incubated at 37°C for 36 h to produce a large number of mycelium. Itraconazole at a final concentration of 2 × MIC was added to the centrifuge tube and incubated for 1 h at 37°C, followed by centrifugation in a centrifuge (3,000 r, 15 min). The supernatant was put into a UV–Vis spectrophotometer for detection at 262 nm (Ohkubo and Osanai, 2005). In addition, the combined drug treatment group was incubated with 2 × MIC itraconazole and 2 × MIC domiphen for 1 h at 37°C, and the subsequent operations were the same as above. The OD values of the two groups were analyzed to determine whether the intracellular itraconazole content was changed after adding domiphen.

### 2.13 Propidium iodide staining cell membranes

200 μL of diluted *A. fumigatus* solution was added to sterile 24-well plates and incubated for 18 h. Subsequently, the fungal solution was aspirated, and the plate was washed three times with PBS, followed by the addition of 400 μL of drug concentration of 2 × MIC of itraconazole and domiphen to each well (200 μL was added to each of the combined drug treatment groups). After 1 h of incubation, the drugs were aspirated and washed on the plates, paraformaldehyde was added for 15 min of fixation, and 200 μL of fluorescent dye PI (final concentration of 40 μM) was treated for 15 min, then observed by fluorescence microscopy. The fluorescent dye was detected with excitation at 488 nm, and emission at 500~550 nm (Yi et al., 2023).

### 2.14 Biofilm clearance

100 μL of the diluted fungal suspension was added to a 96-well plate and incubated at 37°C for 24 h to form a biofilm; the fungal solution was aspirated and washed with PBS to remove the adherent pathogenic fungi. Drug combinations of different concentrations were prepared, and 100 μL was absorbed and added to sterile 96-well plates. The culture medium group was served as a blank control. Subsequently, the solution was aspirated and fixed with 100 μL of methanol for 15 min. Then, the plates were stained with crystal violet for 10 min. Finally, the 96-well plates were washed with PBS for three times. The residual biofilm was dissolved with 95% ethanol, and OD values were measured at 620 nm by using a microplate reader. The Biofilm Eradication Concentration (BEC50) refers to the minimum concentration of a compound at which a drug clears a pathogen biofilm to less than 50% (Kirchhoff et al., 2022).

### 2.15 Transmission electron microscopy analysis of the cell wall

The cell walls of *A. fumigatus* Shjt 40 with and without the combined drugs were examined by TEM. After 24 h of incubation, sodium phosphate buffer in 2.5% glutaraldehyde was used to fix mycelia at 4°C. The samples were initially treated with a 1% osmium acid solution for a duration of 1–2 h. Subsequently, the samples underwent three rinses with a 0.1 M, pH 7.0 phosphate buffer for 15 min each. This was followed by dehydration using ethanol solutions of varying concentrations for 15 min each, and then twice with 100% ethanol for 20 min each. Finally, the samples were subjected to pure acetone for a duration of 20 min. The samples were then treated with a mixture of encapsulant and acetone. The samples underwent treatment with a combination of an embedding agent and acetone. Subsequently, the permeabilized samples were embedded and subjected to overnight heating at a temperature of 70°C. Following this, the samples were sectioned using an ultrathin slicer, resulting in sections measuring 70–90 nm. These sections were then stained with uranyl acetate and lead citrate before being examined under transmission electron microscopy at an acceleration voltage of 80 kV (Sun C. et al., 2022).

## 3 Results

### 3.1 Antimicrobial effects of itraconazole and domiphen on azole-resistant *Aspergillus fumigatus* and biocompatibility of drugs

**Figure 1:**
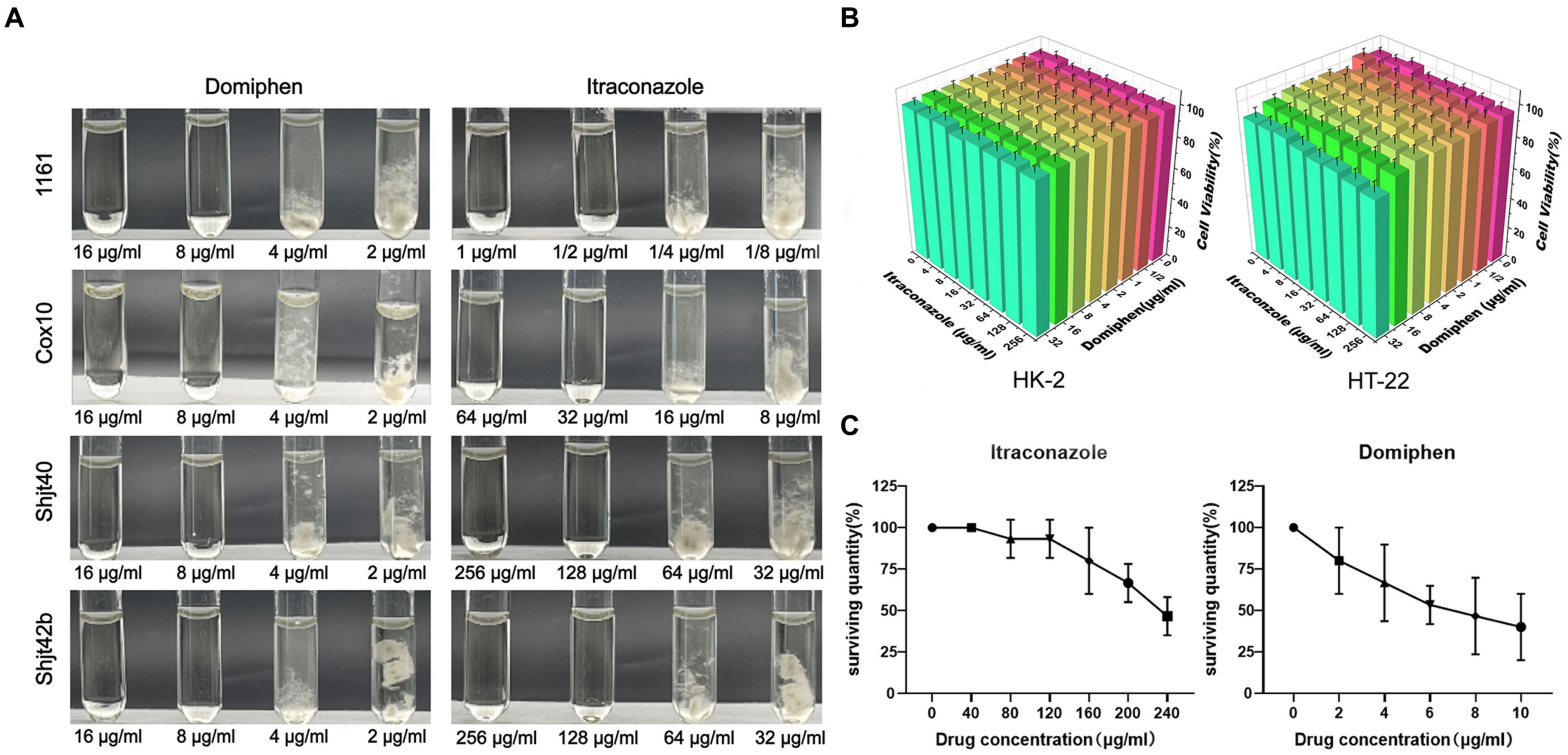
Antimicrobial capacity and biocompatibility of the drug. **(A)** MICs of domiphen and itraconazole against four strains of *Aspergillus fumigatus*. In each group of pictures, the first two test tubes are clarified MM solutions, the last two test tubes are turbid MM solutions containing the mycelium of *A. fumigatus*. **(B)** Cytotoxicity of itraconazole and domiphen. The cells used were HK-2 (human renal cortex proximal convoluted tubule epithelial cells) and HT-22 (mouse hippocampus nerve cells). **(C)** Acute toxicity experiments with itraconazole and domiphen. Both drugs have low toxicity to zebrafishes.

The MICs of domiphen against wild-type *A. fumigatus* (1161) and the three azole-resistant *A. fumigatus* (Cox 10, Shjt 40 and Shjt 42b) were all 8 μg/mL, indicating that all four strains had the same MICs for domiphen, which is likely due to its ability to remove biofilms for azole-resistant strains. Meanwhile, the MICs of itraconazole against *A. fumigatus* 1161 were 1/2 μg/mL, 32 μg/mL for Cox 10, and 128 μg/mL for Shjt 40 and Shjt 42b, suggesting that the azole-resistant *A. fumigatus* including Cox 10, Shjt 40 and Shjt 42b, exhibited strong resistance to itraconazole, showing MICs that were 64, 256, and 256 times, respectively, higher than that of the wild type (Figure 1A). To test the biosafety of these two agents, we performed drug cytotoxicity tests. As shown in Figure 1B, at most concentrations of drug combinations, HT-22 cells and HK-2 cells both had survival rates of around 100%. When the concentration of domiphen is high, it will produce some cytotoxicity to HT-22 cells, and the cell survival rate is about 83%–87%. We also conducted pharmacological tests for acute toxicity. In acute toxicity testing, zebrafish survival decreases as drug concentrations increase. A drug’s Lethal Concentration 50 (LC50) is determined when survival is less than 50%. For itraconazole, the LC50 is greater than or equal to 240 μg/mL, and for domiphen, the LC50 is greater than or equal to 10 μg/mL (Figure 1C). Both cck-8 tests and acute toxicity tests suggested that both itraconazole and domiphen showed minimal cytotoxicity and good biocompatibility.

### 3.2 *In vitro* synergistic antimicrobial properties of domiphen and itraconazole against *Aspergillus fumigatus*

**Figure 2:**
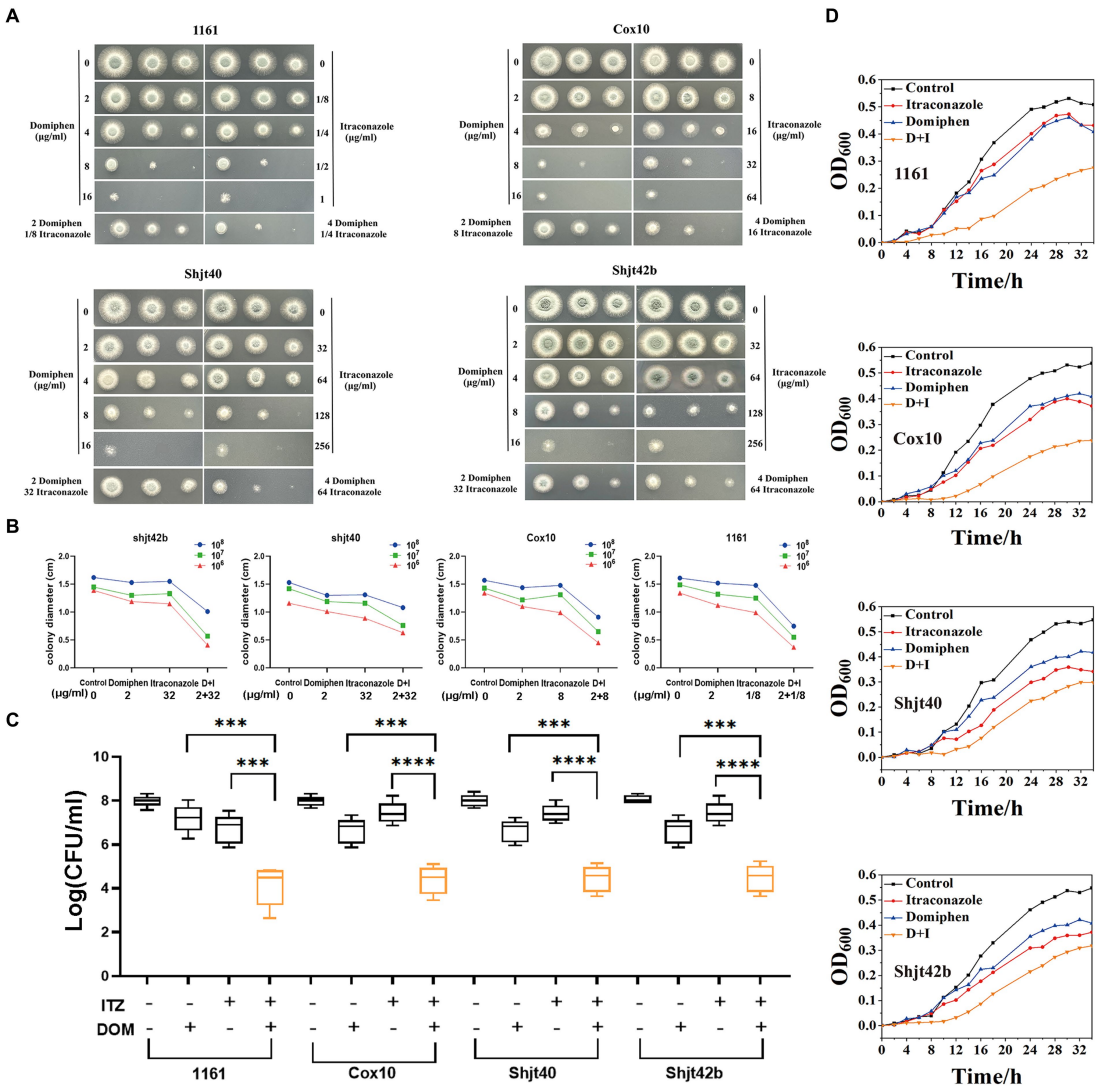
*In vitro* synergistic antimicrobial activity of drug combinations against *Aspergillus fumigatus*. **(A)** Plate spotting of domiphen and itraconazole against four strains of *A. fumigatus*. The concentration of *A. fumigatus* in the first, second and third columns was 10^8^, 10^7^, and 10^6^ CFU/mL, respectively. **(B)** Size of colony diameter after drug inhibition. Combination therapy for *A. fumigatus* is more effective than monotherapy. D+I, Domiphen+Itraconazole. **(C)** Determination of the antimicrobial activity of domiphen and itraconazole against four *A. fumigatus* strains. “+” represents the administration of 1/2 × MIC concentration of the single drug; or 1/4 × MIC concentration of combined drugs. “−” represents administration of the same volume of sterile water. After 36 h of treatment, the number of CFU was measured. Data from six replicates were collected to obtain mean log CFU values. Statistical analysis was performed to determine significant differences between the combined treatments and the single compounds. ^***^p < 0.001; ^****^p < 0.0001 according to unpaired two-tailed *t*-test. **(D)** Growth curves of domiphen, itraconazole and the combination against four strains of *A. fumigatus*. D+I, Domiphen+Itraconazole.

In order to detect the synergistic antimicrobial activity of drugs in solid media, fungal solution was inoculated into solid plates containing a series of drugs concentrations. As shown in Figure 2A, the suspension of *A. fumigatus* was inoculated into YAG solid media with different drug concentrations in the order of 1 × 10^8^ CFU/mL, 1 × 10^7^ CFU/mL, and 1 × 10^6^ CFU/mL, showing round colonies of different sizes. A significant decreasing change in colony diameter was observed with increasing drug concentrations. The colony diameters of the four *Aspergillus* strains on the plates containing itraconazole or domiphen were significantly larger than those of the plates with the combined drug (Figures 2A,B). These results showed that the combination of itraconazole and domiphen has a synergistic antimicrobial effect against *A. fumigatus* on solid media.

In order to investigate the synergistic antimicrobial activity of the drugs in the liquid medium, we performed an assay for inhibition activity. As observed in Figure 2C, the addition of 1/2 × MIC itraconazole or domiphen had a slight inhibitory effect on all four strains of *A. fumigatus*, resulting in a decrease in the biomass in the fungal suspension. Moreover, the addition of combined drugs containing 1/4 × MIC itraconazole and 1/4 × MIC domiphen significantly reduced the fungal biomass. Analysis by unpaired two-tailed t-test showed a significant difference between the single and combined drug-sensitivity groups (*p* < 0.001). These results indicated that the drug combination had a synergistic antimicrobial effect on *A. fumigatus* in liquid media.

To explore the synergistic inhibition of *A. fumigatus* by the drug combination at various times, we detected growth curves of *A. fumigatus*. Figure 2D showed that the growth rate of all four *A. fumigatus* strains were somewhat inhibited by the addition of itraconazole and domiphen treatment, respectively, and the inhibition effect was more apparent in the combined treatment group. The experiments clearly showed the growth of *A. fumigatus* at different moments and the synergistic inhibition of this fungus by the combination at all periods of growth.

In order to further confirm the synergistic antimicrobial effect of these two drugs, we conducted fractional inhibitory concentration (FIC) tests. The square microdilution method determined the combined susceptibility of itraconazole and domiphen at different concentrations to the four *A. fumigatus* strains. The results of the FIC tests can be expressed as synergistic (FIC ≤ 0.5), irrelevant effects (0.5 < FIC ≤ 4), and antagonistic (FIC ≥ 4; Xu et al., 2018). As shown in Table 1, the drug combination had an FIC of 0.25 for 1161, 0.375 for Cox 10 and 0.5 for Shjt 40 and Shjt 42b, demonstrating that the combination of itraconazole and domiphen had a synergistic effect on all four *A. fumigatus* strains.

**Table 1:** Drug susceptibility test of itraconazole and domiphen combined.

Strains	MIC (ppm)		MIC’ (ppm)		FIC
	Itz	Dom	Itz	Dom	
1161	1/2	8	1/16	1	0.25
Cox10	32	8	4	2	0.375
Shjt40	128	8	32	2	0.5
Shjt42b	128	8	32	2	0.5

Results of the combined drug sensitivity test of domiphen and itraconazole. FIC = MIC’(itraconazole)/MIC(itraconazole) + MIC’(domiphen) + MIC(domiphen). MIC, the MIC of single drug; MIC’, the MIC of combined drug.

### 3.3 *In vivo* synergistic antimicrobial properties of domiphen and itraconazole against *Aspergillus fumigatus*

**Figure 3:**
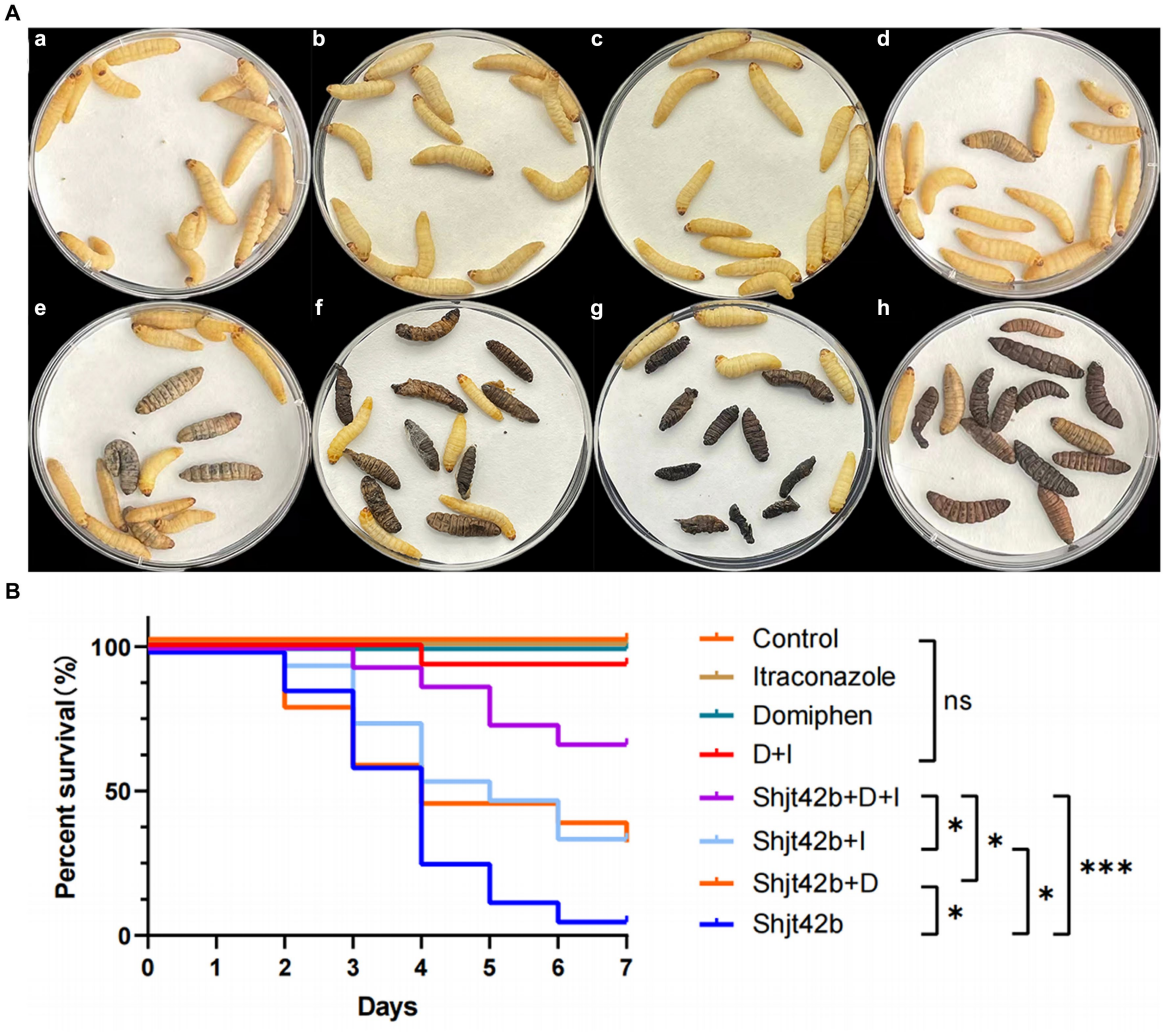
*In vivo* effects of drug combinations on *Aspergillus fumigatus* antifungal activity. **(A)** Day 7 of an *in vivo* experiment with *Galleria mellonella* larvae. (a) injected with saline, (b) injected with itraconazole, (c) injected with domiphen, (d) injected with combination drug, (e) injected with fungus and combination drug, (f) injected with fungus and itraconazole, (g) injected with fungus and domiphen, (h) injected with fungus (Shjt 40). **(B)** Survival curve of *G. mellonella* larvae. I, Itraconazole; D, Domiphen; ^*^p <  0.05; ^***^p < 0.001; ns, *p* > 0.05 according to log rank analysis.

Different fluids (saline, drugs, fungal solutions) were injected into *G. mellonella* larvae in order to assess the antifungal activity of the drugs through their survival rate. The results, as depicted in Figure 3A, indicate that no larvae perished in the saline injection or single drug injection groups. However, one larva died in the combination group, while 14 larvae succumbed to the fungal solution treatment. Furthermore, 10 larvae died in the domiphen or itraconazole treatment group infected with the fungal, individually, whereas only 5 larvae perished in the combined drug treatment group infected with the fungal. The results showed that the mortality rate of the fungal-infected larvae treated with the drug combination was significantly reduced compared to that treated with the drug alone (Figure 3B), indicating a strong *in vivo* synergetic antifungal effect of domiphen and itraconazole.

### 3.4 Transcriptome changes induced by domiphen in *Aspergillus fumigatus*

**Figure 4:**
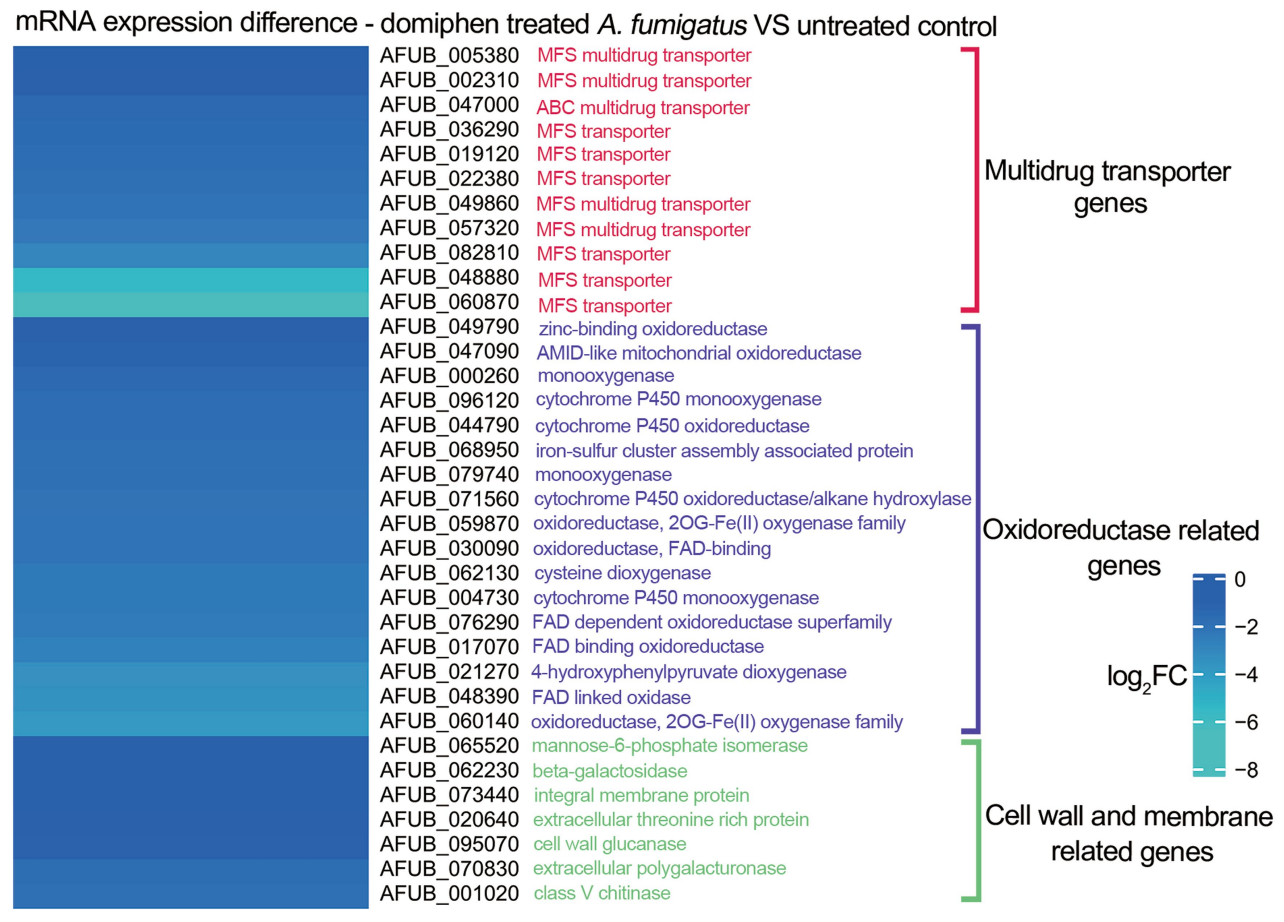
mRNA expression of *Aspergillus fumigatus* after domiphen treatment. The heatmap showing log_2_FC of each genes was generated using MeV (https://webmev.tm4.org/) based on RNA-seq data. Wild-type conidia were collected and incubated in MM for 18 h at 37°C followed by 1 h of domiphen (2 μg/mL) incubation.

To further investigate the potential antimicrobial mechanisms underlying the combined action of the two drugs, we previously conducted a series of mRNA sequencing experiments in which we compared the transcriptional profiles of wild-type strains treated with domiphen to those of non-treated controls (Du et al., 2023). As illustrated in Figure 4, our analysis revealed that, in the domiphen-treated group, the expression levels of certain mRNA transcripts were significantly reduced compared to the control group. Notably, these downregulated transcripts included genes involved in drug efflux, redox reactions, and cell membrane and cell wall remodeling processes. Based on these findings, we postulate that domiphen and itraconazole might exhibit a synergistic antimicrobial effect, potentially arising from the ability of domiphen to inhibit azole drug efflux, oxidoreductase activity, and cell membrane and cell wall remodeling.

### 3.5 Effect of domiphen and itraconazole on intracellular drug retention and biofilm of *Aspergillus fumigatus*

**Figure 5:**
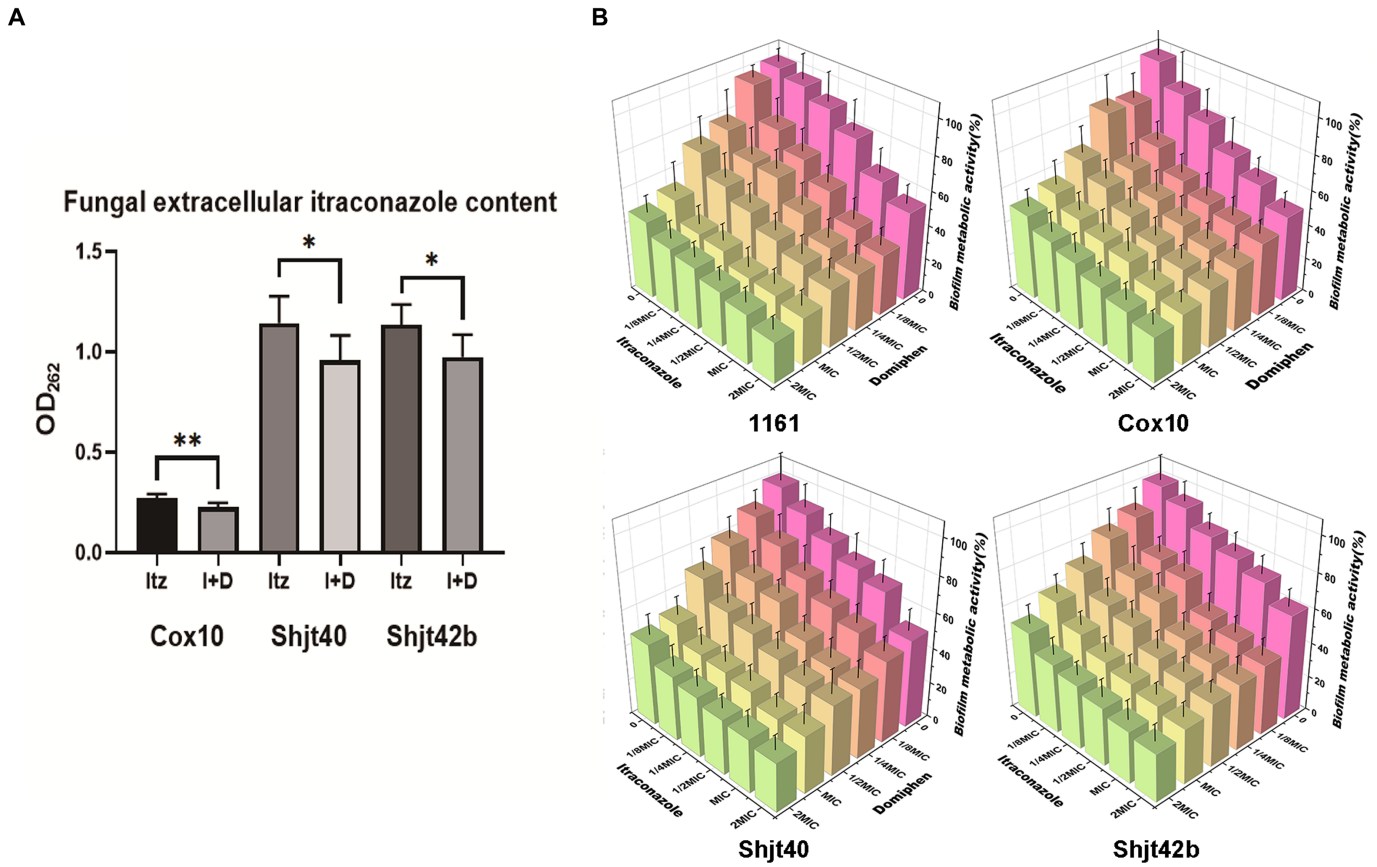
Effect of domiphen and itraconazole on intracellular drug retention and biofilm of *Aspergillus fumigatus*. **(A)** Extracellular itraconazole content in *A. fumigatus* cells treated with itraconazole before and after domiphen treatment indicated as Itz and I+D, respectively. ^*^p < 0.05; ^**^p < 0.01 according to unpaired two-tailed *t*-test. **(B)** Biofilm clearance efficiency of domiphen, itraconazole, and domiphen combined with itraconazole against four *A. fumigatus* strains.

In order to validate the inhibition of the drug efflux system in the mRNA sequencing, we performed drug efflux experiments. As shown in Figure 5A, the three itraconazole-treated resistant strains showed a decrease in extracellular itraconazole content after adding domiphen. Therefore, these results inferred that domiphen could reduce itraconazole efflux from the fungus or enhance itraconazole entry, resulting in higher intracellular itraconazole content and a better antimicrobial effect. Considering that biofilm is an important factor causing drug resistance, we performed biofilm clearance assays. As shown in Figure 5B, by increasing drug concentrations, itraconazole and domiphen were able to clear the biofilm more effectively. The BEC50 of itraconazole against 4 strains of *A. fumigatus* was 2 × MIC, and the BEC50 of domiphen against 4 strains of *A. fumigatus* was 1 × MIC. And it can be found that when the two drugs were combined, the ability to clear the biofilm was enhanced. The experimental results showed that both of these drugs have the ability to remove biofilm and domiphen had a more potent inhibitory effect on the biofilm than itraconazole. Our findings suggest that domiphen, with its strong anti-biofilm properties, may disrupt the biofilm structure and create channels for itraconazole to penetrate and exert its antimicrobial effect.

### 3.6 Effect of domiphen and itraconazole on cell membrane and cell wall of *Aspergillus fumigatus*

**Figure 6:**
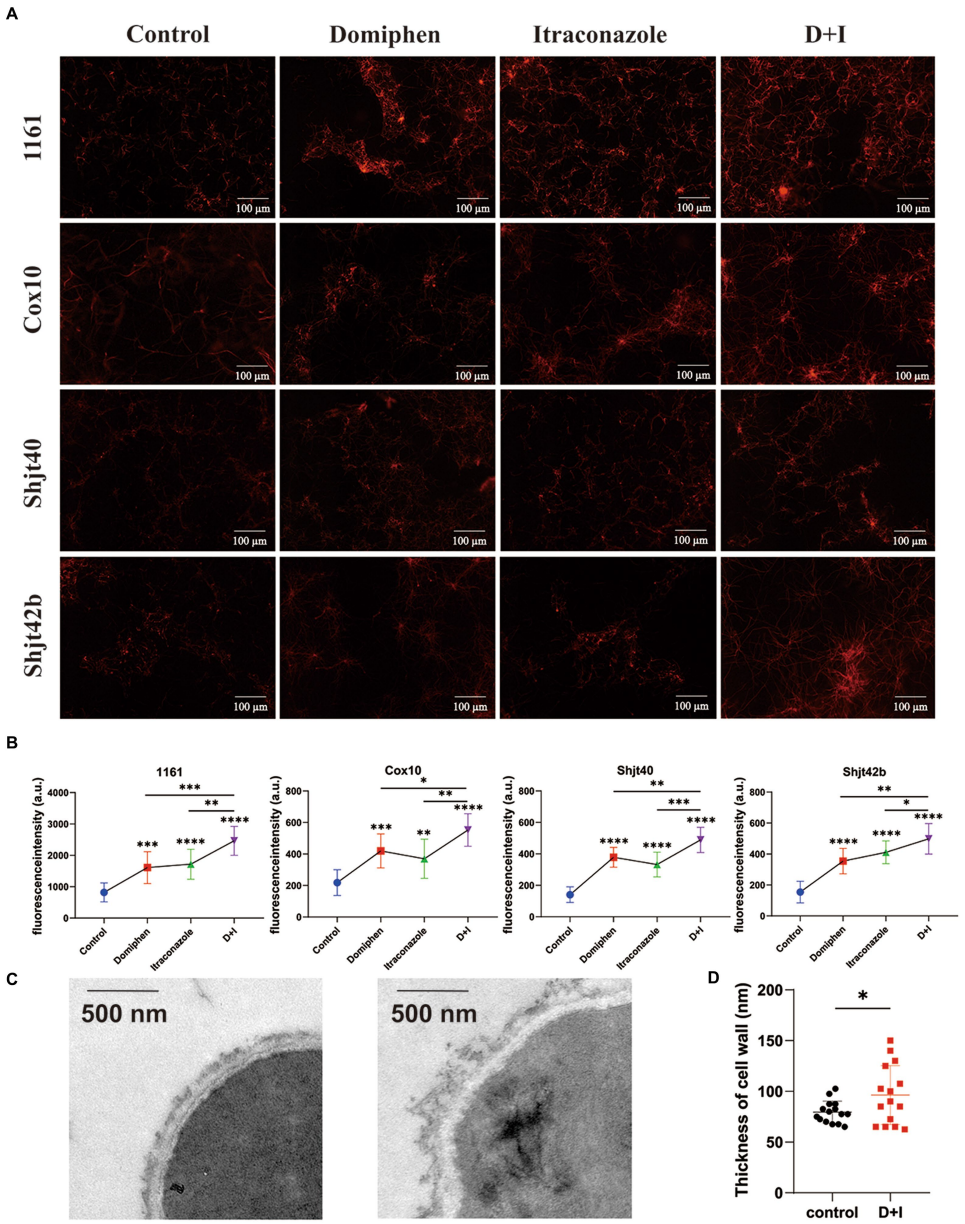
Effect of domiphen and itraconazole on cell membrane of *Aspergillus fumigatus*. **(A)** Propidium Iodide (PI) reagent staining of *A. fumigatus* cells with or without indicated drug treatment. D+I, Domiphen+Itraconazole. **(B)** Fluorescence intensity of drug-treated *A. fumigatus*. D+I, Domiphen+Itraconazole; ^*^p < 0.05; ^**^p < 0.01; ^***^p < 0.001; ^****^p < 0.0001. **(C)** Transmission Electron Microscopy (TEM) of *A. fumigatus* without (left) or with (right) drug treatment. Scale bar = 500 nm. **(D)** Cell wall thickness of *A. fumigatus* with and without combined drug treatment. D+I, Domiphen+Itraconazole; ^*^p < 0.05.

To explore the mRNA sequencing regarding the effects of domiphen on the cell membrane of *A. fumigatus*, we performed propidium iodide (PI) staining experiments. Cell membrane fragmentation will be colored by PI reagent, and fluorescence will be emitted. Brighter fluorescence indicates a greater level of cell membrane fragmentation. As shown in Figures 6A,B, the fluorescence intensities of both the drug-alone group were more significant than that of the control group, and the fluorescence intensities of the combined drug group were more remarkable than that of the drug-alone group, suggesting that the drug combination had a greater ability to disrupt cell membranes or inhibit their production, thus providing a synergistic antimicrobial effect. In order to explore whether this drug combination also has some effect on the cell wall, we did TEM experiments to observe the changes in the cell wall. As shown in Figures 6C,D, the structure of cell wall of *A. fumigatus* after the combination of drugs treatment became more loose, uneven, and thickened compared with untreated control.

### 3.7 Mechanisms of action of domiphen combined with itraconazole against itraconazole-resistant strains of *Aspergillus fumigatus*

**Figure 7:**
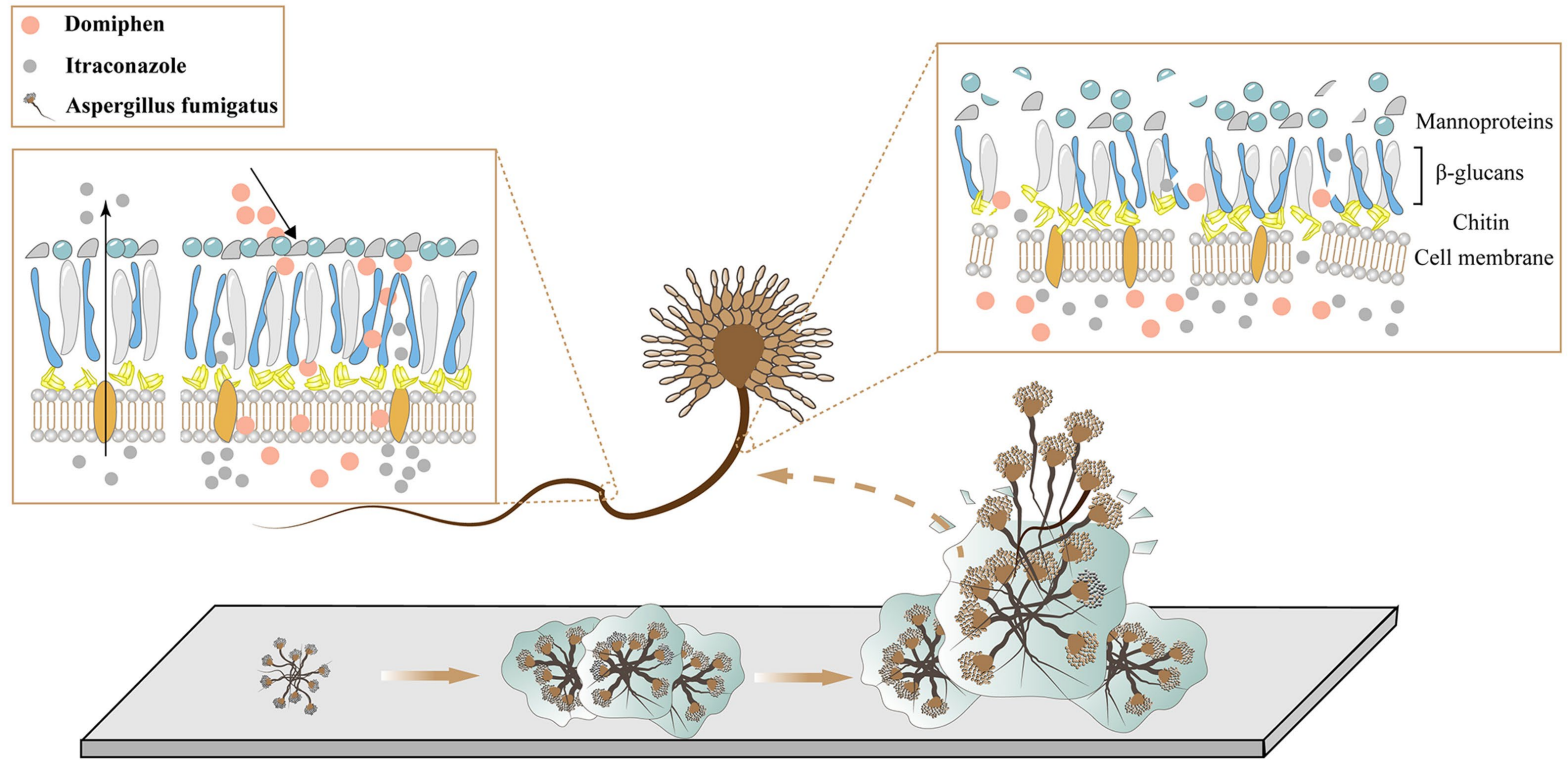
Mechanism of synergistic antimicrobial of domiphen and itraconazole.

As shown in Figure 7, during the growth of fungi, the fungi gather and adhere to each other, forming a biofilm. At this time, the fungus is wrapped in its own secreted polysaccharide, and it is difficult for the drug to invade. Domiphen has the ability to break the biofilm effectively. After the addition of domiphen, the biofilm of the fungus is effectively removed, and itraconazole can then effectively act on the fungus. In addition, the treatment with domiphen resulted in a increase of intracellular itraconazole retention. At the same time, these two drugs prevent the cell membrane of the fungal cells from reforming and change the structure of the cell wall.

## 4 Discussion

*Aspergillus fumigatus* is a significant fungus causing food spoilage, and its colonies are characterized by rapid growth and can overgrow at temperatures of 45°C or higher (Wassano et al., 2020). Although *A. fumigatus* is a weak pathogen, severe and often fatal invasive Aspergillosis (IA) has increased dramatically with the number of immunosuppressed patients, and IA is now a widespread fungal infection worldwide (Douglas et al., 2021). Currently, some azoles can effectively inhibit the growth of *A. fumigatus*. With the heavy use of these drugs, the biofilm of *A. fumigatus* becomes exceptionally resistant, thus making it impossible to eradicate *A. fumigatus* (Nywening et al., 2020; Rivelli Zea and Toyotome, 2022). Despite the increasing number of infections caused by fungal biofilms, only a few new antifungal drugs with antifungal film activity, such as voriconazole and fluconazole, have been developed in the past decades (Ghobadi et al., 2022). However, the high cost of voriconazole and the disadvantage of the low antifungal efficacy of fluconazole has left a dearth of antifungal drugs in this field. At present, many studies have shown that the use of drugs in combination with antifungal therapy can effectively reduce the side effects and resistance of single antifungal drugs (Borba-Santos et al., 2021; Lee et al., 2021; Zhu et al., 2023), and it has important value in the treatment of resistant fungi. Therefore, in this study, the mechanism of the strong antifungal performance of itraconazole combined with domiphen against drug-resistant *A. fumigatus* was provided. Moreover, this combination reduced the dosage of the drug, which reduced not only the cost but also the toxicity of the drug, providing solid theoretical support for developing specific antifungal drugs for *A. fumigatus*.

In order to explore the antimicrobial superiority of this drug combination. Herein, sufficient synergistic antifungal experiments were performed. The MIC and FIC values of the four strains were measured and gradually verified that the drug combination was synergistic (FIC ≤ 0.5) for all four strains. The size change of fungal diameter after inhibition could be observed visually by plate spotting test. Inhibition activity and growth curve assay were performed to identify the synergistic antifungal ability from multiple perspectives. All of the above-mentioned experiments could demonstrate that when combined with domiphen and itraconazole, it has better antifungal efficacy than drug treatment alone. We tried to unravel the mystery behind the apparent phenomenon and investigate clearly the synergistic antifungal mechanism of this drug combination. Thus, We combined previous studies and used RNA-seq to find and screen for genetic alterations in *A. fumigatus* after treatment with domiphen. For example, the expression of genes related to the control of drug efflux, oxidative stress, and the synthesis of cell membranes and cell walls is decreased. Therefore, we carried out further studies on the antifungal mechanisms.

The formation of biofilm is a unique biological phenomenon for microorganisms to protect themselves. It has been reported that the formation of fungal biofilm is positively correlated with its drug resistance (Vuotto et al., 2017). In our previous study, we mentioned that domiphen has the ability to remove the biofilm of *Candida albicans* (Hu et al., 2021). In order to investigate whether domiphen and itraconazole can also be effective in removing biofilm of *A. fumigatus*, we conducted relevant experiments and concluded that both itraconazole and domiphen can effectively remove biofilm, and the anti-biofilm activity of domiphen is more superior than itraconazole. Therefore, we speculate that domiphen is more likely to break through the protection of biofilm and act on some sites of fungi first, thus facilitating itraconazole to enter the fungal cells and strengthen the antifungal performance. Besides, itraconazole has broad-spectrum antifungal activity, and studies have shown that the earliest ultrastructural changes after itraconazole treatment include abnormalities in the plasma membrane, cell wall, and cytoplasmic vesicles (Borgers and Van de Ven, 1989). The main mechanism is the inhibition of fungal ergosterol synthesis, which disrupts cell membrane production. In order to investigate whether domiphen could enhance the disruption of cell membrane by itraconazole, we did PI staining experiments. It was found that the fluorescence emitted by the combined drug group was more intense, indicating that the cell membrane rupture was greater and itraconazole combined with domiphen played a stronger role in disrupting the production and normal metabolism of the cell membrane. Meanwhile, we examined the cell wall structure of *A. fumigatus* by TEM and found that the cell wall was generally thickened and uneven in shape after the combined drug treatment, which might be caused by the internal structure becoming loose. Drug efflux pump is a kind of protein which exists in the cell membrane of microorganism. Many studies have found that many microorganisms can pump antifungal drugs out of the cell through the efflux pump system, thus reducing the concentration of drugs in the fungi and leading to drug resistance (Galocha et al., 2020; Perez-Cantero et al., 2020). In the present study, we examined the intracellular itraconazole content of the fungus with and without domiphen and found that the fungal cells contained more itraconazole drug in the group with domiphen. Hence, our findings suggest that the inhibition of the drug efflux pump gene by domiphen may potentially facilitate the intracellular accumulation of itraconazole, leading to effective eradication of *A. fumigatus*. It is important to note that the observed increase in itraconazole content within *A. fumigatus* cells could also be attributed to alterations in permeability resulting from the disruption of biofilms, cell walls, and cell membranes, which serve as external protective barriers.

To ascertain the potential issue of drug biocompatibility, we undertook pertinent investigations, including cck-8 experiments and zebrafish mortality experiments, which revealed the drug’s minimal toxicity. Furthermore, *in vivo* experiments employing *G. mellonella* larvae demonstrated the drug combination’s biosafety and robust *in vivo* antifungal efficacy, leading to a significant reduction in mortality among larvae infected with *A. fumigatus*.

## 5 Conclusion

In summary, as shown in Figure 7, we speculate that the overall drug mechanism of action is that domiphen alters the permeability of the biofilm, cell membrane, cell wall and other structures of *A. fumigatus*, thereby changing the distribution of itraconazole in *A. fumigatus*, allowing itraconazole to act better on *A. fumigatus* and provide an efficient antifungal effect. At the same time, domiphen and itraconazole were also less toxic. This provides effective and rational guidance for developing antifungal agents against *A. fumigatus* and avoiding the potential dangers of conventional antimicrobials.

## Patents

The authors report a patent CN116077499A licensed to Wenlong Du, Huan Yang, Yufan Gu, Jia-nan Wu, Yipeng Pang, Zhiqiang He, Jiawei Song, Linjie Xu, Da Zong and Yi Liu. No other competing interests exist.

## Data availability statement

The datasets presented in this study can be found in online repositories. The names of the repository/repositories and accession number(s) can be found at: BioProject, PRJNA856787.

## Ethics statement

The manuscript presents research on animals that do not require ethical approval for their study.

## Author contributions

HY: Conceptualization, Methodology, Writing – original draft. YG: Conceptualization, Data curation, Methodology, Writing – review & editing. ZH: Formal analysis, Writing – review & editing. J-NW: Investigation, Validation, Writing – review & editing. CW: Validation, Writing – review & editing. YX: Investigation, Writing – review & editing. YYo: Visualization, Writing – review & editing. YYa: Formal analysis, Writing – review & editing. XZ: Data curation, Writing – review & editing. YL: Resources, Supervision, Writing – review & editing. WD: Funding acquisition, Project administration, Resources, Supervision, Writing – review & editing.
